# The “Rorick System of Rectal Surgery”

**Published:** 1889-03

**Authors:** 


### THE "RORICK SYSTEM OF RECTAL SURGERY."

We are much chagrined to learn that some of our subscribers—seeing the above named article advertised in this JOURNAL, purchased the outfit, and found themselves as one expresses it, "badly bit," and another, "victimized" by it. Dr. T. M. Wilson, of Kosse, says, "I wrote him, [the proprietor] as advertised in your JOURNAL, and this little pamphlet which I send you, is the result; you will see that it is a regular quack concern. On receiving it I wrote to Dr. —— [name withheld for the present only because we have not the Doctor's permission to use it, as we have in Dr. Wilson's case.—ED.] and he acknowledged it a humbug, and that he was victimized. The instruments should not cost over $10 and the "treatise" $2. Yet he will not sell them separately, not for less than $50. Dr. —— says the "treatise" is only a little pamphlet and contains nothing of value or importance. We naturally look to our teachers for protection against quackery."

We regret exceedingly to hear such reports. The advertisement we saw in many of the leading Journals, and we presumed it was all right, hence when it was tendered the JOURNAL we accepted it. The contract has long since expired and the "ad." has disappeared from our pages. We tender Dr. Wilson and his friend our sincere apologies, and we will endeavor to prevent the appearance of similar advertisements in our columns in future.

---

THE GOVERNOR OF LOUISIANA has raised a hornets' nest about his ears by the removal of Dr. Aby—long time quarantine officer at New Orleans, without cause, as alleged—and the appointment of Dr. W. G. Austin.

